# Supplementary material for: Heat adaptation of phage T7 under an extended genetic code
Source: Virus Evol. 2021 Dec 1;7(2):veab100. doi: 10.1093/ve/veab100 (PMC8923235; doi:10.1093/ve/veab100)
Supplement: veab100_Supp [file veab100_supp.zip › Sequencing_Analysis.zip!/Sequencing_Analysis/A2/evidence/RA_24.html]

BRESEQ :: Evidence


| Read alignment evidence... | | | | | | | | | | | |
| --- | --- | --- | --- | --- | --- | --- | --- | --- | --- | --- | --- |
|  | seq id | position | | ref | new | freq | score (cons/poly) | reads | annotation | genes | product |
| \* | NC\_001604.1 | 26,933 | 0 | A | G | 36.7% | 217.8 / inf | 411 | intergenic (–/–) | *–/–* | –/– |
| Reads supporting (aligned to +/- strand):  **ref** base A (102/158);  **new** base G (59/92);  **total** (161/250) | | | | | | | | | | | |
| Fisher's exact test for biased strand distribution *p*-value = 1.00e+00 | | | | | | | | | | | |
| Kolmogorov-Smirnov test that lower quality scores support variant *p*-value = 6.72e-02 | | | | | | | | | | | |

Only 100 of 412 total aligned reads displayed.  

`ATTCGATACACGATTCCTAGTGGAACATACAACGATGACACATTCACTACCTCTATTCATATTCCAACAATTTATGGTGCAAACTTCGGGAGGGGCAAAATCACTGTATTGGAGCCTGATGGTAAGATAACCGTGTTTGAGCAACCTACGGCTGGGTGGAATAGCGACCCTTGGCTGAGACTCAGCGGTAACTTGGAGGGACGCATGGTGTACATTGGGTTCAACATTAACTTCGTATATGAGTTCTCTAAGTTCCTCATCAAGCAGACTGCCGACGACGGGTCTACCTCCACGGAAGACATTGGGCGCTTACAGTTACGCCGAGCGTGGGTTAACTACGAGAACTCTGGTACGTTTGACATTTATGTTGAGAACCAATCGTCTAACTGGAAGTACACAATGGCTGGTGCCCGATTAGGCTCTAACACTCTGAGGGCTGGGAGACTGAACTTAGGGACCGGACAATATCGATTCCCTGTGGTTGGTAACGCCAAGTTCAACACTGTATACATCTTGTCAGATGAGACTACCCCTCTGAACATCATTGGGTGTGGCTGGGAAGGTAACTACTTACGGAGAAGTTCCGGTATTTAATTAAAT  >  NC_001604.1/26633‑27232`  

                                                                                                                                                                                                                                                                                                            `|`                                                                                                                                                                                                                                                                                                             
       `acacGATTCCTAGTGGAACATACAACGATGACACATTCACTACCTCTATTCATATTCCAACAATTTATGGTGCAAACTTCGGGAGGGGCAAAATCACTGTATTGGAGCCTGATGGTAAGATAACCGTGTTTGAGCAACCTACGGCTGGGTGGAATAGCGACCATTGGCTGAGACTCAGCGGTAACTTGGAGGGACGCATGGTGTACATTGGGTTCAACATTAACTTCGTATATGAGTTCTCTAAGTTCCTCATCAAGCAGACTGCCGACGACGGGTCTACCTCCACGGAAGACGTTGGgcg                                                                                                                                                                                                                                                                                                      >  1:210134/1‑301 (MQ=255)`  
             `ttCCTAGTGGAACATACAACGATGACACATTCACTACCTCTATTCATATTCCAACAATTTATGGTGCAAACTTCGGGAGGGGCAAAATCACTGTATTGGAGCCTGATGGTAAGATAACCGTGTTTGAGCAACCTACGGCTGGGTGGAATAGCGACCCTTGGCTGAGACTCAGCGGTAACTTGGAGGGACGCATGGTGTACATTGGGTTCAACATTAACTTCGTATATGAGTTCTCTAAGTTCCTCATCAAGCAGACTGCCGACGACGGGTCTACCTCCACGGAAGACATTGGGCGCTTACa                                                                                                                                                                                                                                                                                                >  1:625392/1‑301 (MQ=255)`  
                `cTAGTGGAACATACAACGATGACACATTCACTACCTCTATTCATATTCCAACAATTTATGGTGCAAACTTCGGGAGGGGCAAAATCACTGTATTGGAGCCTGATGGTAAGATAACCGTGTTTGAGCAACCTACGGCTGGGTGGAATAGCGACCCTTGGCTGAGACTCAGCGGTAACTTGGAGGGACGCATGGTGTACATTGGGTTCAACATTAACTTCGTATATGAGTTCTCTAAGTTCCTCATCAAGCAGACTGCCGACGACGGGTCTACCTCCACGGAAGACATTGGGCGCTTACAGtt                                                                                                                                                                                                                                                                                             <  1:16095/301‑1 (MQ=255)`  
                    `tgggcgcggagatgtgaataagagacagaCCTCTATTCATATTCCAACAATTGATGGTGCAATCTTCGGGAGGGGAAAAATCACTGTATTGGAGCCTGATGTTAAGATAACCGTGTTTGAGCAACCTACGGCTGGGTGGAATAGCGACCCTGGGCTGAGACTCAGCGGTAACTTTGAGGGACGCATGGTGTACATTGGGTTCAACATTAACTTCGTAGATGAGTTCTCTAAGTTCCTCATCAAGCAGACTTCCGACGACGGGTCTACCTCCACGGAAGACATTGGGCGCTTACAGTTACGc                                                                                                                                                                                                                                                                                         <  1:655425/273‑1 (MQ=255)`  
                          `aTACAACGATGACACATTCACTACCTCTATTCATATTCCAACAATTTATGGTGCAAACTTCGGGAGGGGCAAAATCACTGTATTGGAGCCTGATGGTAAGATAACCGTGTTTGAGCAACCTACGGCTGGGTGGAATAGCGACCCTTGGCTGAGACTCAGCGGTAACTTGGAGGGACGCATGGTGTACATTGGGTTCAACATTAACTTCGTATATGAGTTCTCTAAGTTCCTCATCAAGCAGACTGCCGACGACGGGTCTACCTCCACGGAAGACATTTGGCGCTTACAGTTACGCCGAgtg                                                                                                                                                                                                                                                                                   >  1:774752/1‑299 (MQ=255)`  
                             `cAACGATTACACATTCACTACCTCTATTCATATTCCAACAATTTATGGTGCAAACTTCGGGAGGGGCAAAATCACTGTATTGGAGCCTGATGGTAAGATAACCGTGTTTGAGCAACCTACGGCTGGGTGGAATAGCGACCCTTGGCTGAGACTCAGCGGTAACTTGGAGGGACGCATGGTGTACATTGGGTTCAACATTAACTTAGTATATGAGTTCTCTAAGTTCCTCATCAAGCAGACTGCCGACGACGGGTCTACCTCCACGGAAGACATTGGGCGCTTACAGTTACGCCGAGCGTgg                                                                                                                                                                                                                                                                                >  1:645422/1‑301 (MQ=255)`  
                              `aaCGATGACACATTCACTACCTCTATTCATATTCCAACAATTTATGGTGCAAACTTCGGGAGGGGCAAAATCACTGTATTGGAGCCTGATGGTAAGATAACCGTGTTTGAGCAACCTACGGCTGGGTGGAATAGCGACCCTTGGCTGAGACTCAGCGGTAACTTGGAGGGACGCATGGTGTACATTGGGTTCAACATTAACTTCGTATATGAGTTCTCTAAGTTCCTCATCAAGCAGACTGCCGACGACGGGTCTACCTCCACGGAAGACATTGGGCGCTTACAGTTACGCCGAGCGTggg                                                                                                                                                                                                                                                                               <  1:733453/301‑1 (MQ=255)`  
                              `aaCGATGACACATTCACTACCTCTATTCATATTCCAACAATTTATGGTGCAAACTTCGGGAGGGGCAAAATCACTGTATTGGAGCCTGATGGTAAGATAACCGTGTTTGAGCAACCTACGGCTGGGTGGAATAGCGACCCTTGGCTGAGACTCAGCGGTAACTTGGAGGGACGCATGGTGTACATTGGGTTCAACATTAACTTCGTATATGAGTTCTCTAAGTTCCTCATCAAGCAGACTGCCGACGACGGGTCTACCTCCACGGAAGACATTGGACGCTTACAGTTACGCCGAGCATggg                                                                                                                                                                                                                                                                               <  1:695434/301‑1 (MQ=255)`  
                                    `gACACATTCACTACCTCTATTCATATTCCAACAATTTATGGTGCAAACTTCGGGAGGGGCAAAATCACTGTATTGGAGCCTGATGGTAAGATAACCGTGTTTGAGCAACCTACGGCTGGGTGGAATAGCGACCCTTGGCTGAGACTCAGCGGTAACTTGGAGGGACGCATGGTGTACATTGGGTTCAACATTAACTTCGTATATGAGTTCTCTAAGTTCCTCATCAAGCAGACTGCCGACGACGGGTCTACCTCCACGGAAGACATTGGGCGCTTACAGTTACGCCGAGCGTGGGTCAACt                                                                                                                                                                                                                                                                         >  1:309722/1‑301 (MQ=255)`  
                                      `cacaTTCACTACCTCTATTCATATTCCAACAATTTATGGTGCAAACTTCTGGAGGGGCAAAATCACTGTATTGGAGCCTGATTGTAAGATAACCGTGTTTGAGCAACCTACGGCTGGGTGGAATAGCGACCCTTGGCTGAGACTCAGCGGTAACTTGGAGGGACGCATGGTGTACATTGGGTTCAACATTAACTTCGTATATGAGTTCTCTAAGTTCCTCATCAAGCAGACTGCCGACGACGGGTCTACCTCCACGGAAGACATTGGGCGCTTAcctgtctcttatacacatctccgagcc                                                                                                                                                                                                                                                                       >  1:382041/1‑275 (MQ=255)`  
                                       `tagatgtgtataagagacagtatTCCAACAATTTATGGTGCAAACTTCGGGAGTGGCAAAATCACTGTATTGGAGCCTGATGGTAAGATAACCGTGTTTGAGCAACCTACGGCTGGGTGGAATAGCGACCCTTGGCTGAGACTCAGCGGTAACTTGGAGGGACGCATGGTGTACATTGGGTTCAACATTAACTTCGTATATGAGTTCTCTAAGTTCCTCATCAAGCAGACTGCCGACGACGGGTCTACCTCCACGGAAGACATTGGGCGCTTACAGTTACGCCGAGCGTGGGTTAACTACg                                                                                                                                                                                                                                                                      <  1:11933/281‑1 (MQ=255)`  
                                          `ttCACTACCTCTATTCATATTCCAACAATTTATGGTGCAAACTTCGGGAGGGGCTAAATCACTGTATTGGAGCCTAATGGTAAGATAACCGTGTTTGAGCAACCTACGGCTGGGTGGAATAGCGACCCTTGGCCGAGACTCAGCGGTAACTTGGAGGGACGCATGGAGTACATTGGGTTCAACAATACCTTCGTATATGAGTTCTCTAAGTTCCTCATCAAGCAGACTGCCGACGTCGGGTCTACCTCCACGGAAGACATTGGGCGCTTACAGTTACGCTGAGCGTGGGTTAACTACgaga                                                                                                                                                                                                                                                                   >  1:353429/1‑301 (MQ=255)`  
                                              `cTATCTCTATTCATATTCCAACAATTTATGGTGCAAACTTCGGGAGGGGCAAAATCACTGTATTGGAGCCTGATGGTAAGATAACCGTGTTTGAGCAACCTACGGCTGGGTGGAATAGCGACCCTTGGCTGAGACTCAGCGGTAACTTGGAGGGACGCATGGTGTACATTGGGTTCAACATTAACTTCGTATATGAGTTCTCTAAGTTCCTCATCAAGCAGACTGCCGACGACGGGTCTACCTCCACGGAAGACATTGGGCGCTTACAGTTACGCCGAGCGTGGGTTAACTACgactgtct                                                                                                                                                                                                                                                               >  1:305193/1‑295 (MQ=255)`  
                                              `cTACCTCTATGCATATCCCAAACATGTATGGTGCAAAGTTCGGGATGGGCAAAAGCACTGTATTGGAGCCTGATGGTAAGATAACCGTGGTTGAGCAACCTACGGCTGTGTGTCATAGCGACCCTTGGCTGAGACTCAGCGGTAACTTGGAGTGACGCATGGTGTACATTGTGTGCAGCATTAACTTCGTATATGAGTTCTCTAAGTTCCTCATCAAGCAGACTGCCGACGACGGGTCTACCTCCACGGAAGACATTGGGCGCTTACAGTTACGCCGAGCGTGGGTTAACTACGAGAActc                                                                                                                                                                                                                                                               <  1:538807/301‑1 (MQ=255)`  
                                                 `ccTCTATTCATATTCCAACAATTTATGGTGCAAACTTCGGGAGGGGCAAAATCACTGTATTGGAGCCTGATGGTAAGATAACCGTGTTTGAGCAACCTACGGCTGGGTGGAATAGCGACCCTTGGCTGAGACTCAGCGGTAACTTGGAGGGACGCATGGTGTACATTGGGTTCAACATTAACTTCGTATATGAGTTCTCTAAGTTCCTCATCAAGCAGACTGCCGACGACGGGTCTACCTCCACGGCAGACATTGGGCGCTTACAGTTACGCCGAGCGTGGGTTAACTACGAGAACTCTgg                                                                                                                                                                                                                                                            >  1:89372/1‑301 (MQ=255)`  
                                                    `ctATTCATATTCCAACAATTTATGGTGCAAACTTCGGGAGGGGCAAAATCACTGTATTGGAGCCTGATGGTAAGATAACCGTGTTTGAGCAACCTACGGCTGGGTGGAATAGCGACCCTTGGCTTAGACTCAGCGGTAACTTGGAGGGACGCATTGTGTACATTGGGTTCAACATTAACTTCGTATATGAGTTCTCTAAGTTCCTCATCAAGCAGACTGCCGACGACGGGTCTACCTCCACGGAAGACGTTGGGCGCTTACAGTTACGCCgctgtctcttatacacatctccgagcccacg                                                                                                                                                                                                                                                         >  1:79190/1‑271 (MQ=255)`  
                                                    `ctATTCATATTCCAACAATTTATGGTGCAAACTTCGGGAGGGGCAAAATCACTGTATTGGAGCCTGATGGTAAGATAACCGTGTTTGAGCAACCTACGGCTGGGTGGAATAGCGACCCTTGGCTGAGACTCAGCGGTAACTTGGAGGGACGCATGGTGTACATTGGGTTCAACATTAACTTCGTATATGAGTTCTCTAAGTTCCTCATCAAGCAGACTGCCGACGCCGGGTCTACCTCAACGGAAGACATTGGGCGCTTACAGTTACGCCGAGCGTgggctgtctcttatacacatctccg                                                                                                                                                                                                                                                         >  1:607412/1‑279 (MQ=255)`  
                                                    `agatgtgtataagggacagttATGGTGCAAACTTCGGGAGGGGCAAAATCACTGTATTGGAGCCTGATGGTAAGATAACCGTGTTTGAGCAACCTACGGCTGGGTGGAATAGCGACCCTTGGCTGAGACTCAGCGGTAACTTGGAGGGACGCATGGTGTACATTGGGTTCAACATTAACTTCGTATATGAGTTCTCTAAGTTCCTCATCAAGCAGACTGCCGACGACGGGTCTACCTCCACGGAAGACGTTGGGCGCTTACAGTTACGCCGAGCGTGGGTTAACTACGAGAACTCTGGTAc                                                                                                                                                                                                                                                         <  1:371227/282‑1 (MQ=255)`  
                                                      `aTTCATATTCCAACAATTTATGGTGCAAACTTCGGGAGTGGCAAAATCACTGTATTGGAGCCTGATGGTAAGATAACCGTGTTTGAGCAACCTACGGCTGGGTGGAATATCTACCCTTGGCTGAGACTCAGCGGTAACTTGGAGGGACGCATGGTGTACATTGGGTTCAACATTAACTTCGTATATGAGTTCTCTAAGTTCCTCATCAAGCAGACTGCCGACGACGGGTCTACCTCCACGGAAGACGTTGGGCGCTTACAGTTACGCCGAGCGTGGGTTAACTACGAGAACTCTGGTACGt                                                                                                                                                                                                                                                       >  1:132183/1‑301 (MQ=255)`  
                                                             `tcgtgggctcggagatgtgtataagagacaGGGGCAAAATCACTGTATTGGAGCCTGATGGTAAGATAACCGTGTTTTAGCAACCTACGGCTGGGTGGAATAGCTACCCTTGGCTGAGACTCAGCGGTAACTTGGAGGGACGCATGGTGTACATTGGGTTCAACATTAACTTCGTATATGAGTTCTCTAAGTTCCTCATCAAGCAGACTGCCGACGACGGGTCTACCTCCACTGAAGACATTGGGCGCTTACAGTTACGCCGAGCGTGGGTTAACTACGAGAACTCTGGTACGTTTGACAt                                                                                                                                                                                                                                                <  1:253186/272‑1 (MQ=255)`  
                                                                            `gTGCAAACTTCGGGAGGGGCAAAATCACTGTATTGGAGCCTGATGGTAAGATAACCGTGTTTGAGCAACCTACGGCTGGGTGGAATAGCGACCCTTGGCTGAGACTCAGCGGTAACTTGGAGGGACGCATGGTGTACATTGGGTTCAACATTAACTTCGTATATGAGTTCTCTAAGTTCCTCATCAAGCAGACTGCCGACGACGGGTCTACCTCCACGGAAGACATTGGGCGCTTACAGTTACGCCGAGCGTGGGTTAACTACGAGAACTCTGGTACGTTTGACAtctgtctcttatacac                                                                                                                                                                                                                                 >  1:33323/1‑286 (MQ=255)`  
                                                                              `gCAAACTTCGGGAGGGGCAAAATCACTGTATTGGAGCCTGATGGTAAGATAACCGTGTTTGAGCAACCTACGGCTGGGTGGAATAGCGACCCTTGGCTGAGACTCAGCGGTAACTTGGAGGGACGCATGGTGTACATTGGGTTCAACATTAACTTCGTATATGAGTTCTCTAAGTTCCTCATCAAGCAGACTGCCGACGACGGGTCTACCTCCACGGAAGACATTGGGTGCTTACAGTTACGCCGAGCGTTGGTTAACTACGAGAACTCTGGTACGTTTGACATTTATGTTGAGAACCAAt                                                                                                                                                                                                                               <  1:561439/301‑1 (MQ=255)`  
                                                                                         `gAGGGGCAAAATCACTGTATTGGGGCTTGATGGTAAGATAACCGTGTTTGAGCAACCTACGGCTGGGTGGAATAGCGACCATTGGCTGAGACTCAGCGGTAACTTGGAGGGACGCATGGTGTACATTGGGTTCAACATTAACTTCGTATATGAGTTCTCTAAGTTCCTCATCAAGCAGACTGCCGACGACGGGTCTACCTCCACGGAAGACGTTGGGCGCTTACAGTTACGCCGAGCGTGGGTTAACTACGAGAACTCTGGTACGTTTGACATTTATGTTGAGAACCAATCGTCTAACTgg                                                                                                                                                                                                                    <  1:274033/301‑1 (MQ=255)`  
                                                                                                    `tCACTGTATTGGAGCCTGATGGTAAGATAACCGTGTTTGAGCAACCTACGGCTGGGTGGAATAGCGACCCTTGGCTGAGACTCAGCGGTAACTTGGAGGGACGCATGGTGTACATTGGGTTCAACATTAACTTCGTATATGAGTTCTCTAAGTTCCTCATCAAGCAGACTGCCGACGACGGGTCTACCTCCACGGAAGACATTGGGCGCTTACAGTTACGCCGAGCGTGGGTTAACTACGAGAACTCTGGTACGTTTGACATTTATGTTGAGAACCAATCGTCTAACTGGAAGTACACAAt                                                                                                                                                                                                         <  1:719335/301‑1 (MQ=255)`  
                                                                                                       `cgtgggatcggagatgtgtataagagacagtgtTTGAGCAACCTACGGCTGGGTGGAATAGCGACCCTTGGCTGAGACTCAGCGGTAACTTGGAGGGACGAATTGTGTACATTGGGTTCAACATTAACTTCGTATATGAGTTCTCTAAGTTCCTCATCAAGCAGACTGCCGACGACGGGTCTACCTCCACGGAAGACGTTGGGCGCTTACAGTTACGCCGAGCGTGGGTTAACTACGAGAACTCTGGTACGTTTGACATTTATGTTGAGAACCAATCGTCTAACTGGAAGTACACAATGGc                                                                                                                                                                                                      <  1:111041/272‑1 (MQ=255)`  
                                                                                                         `gTATTGGAGCCTGATGGTAAGATAACCGTGTTTGAGCAACCTACGGCTGGGTGGAATAGCGACCCTTGGCTGAGACTCAGCGGTAACTTGGAGGGACGCATGGTGTACATTGGGTTCAACATTAACTTCGTATATGAGTTCTCTAAGTTCCTCATCAAGCAGACTGCCGACGACGGGTCTACCTCCACGGAAGACGTTGGGCGCTTACAGTTACGCCGAGCGTGGGTTCACTACGAGAACTCTGGTACGTTTGACATTTATGTTTAGAACCAATCGTCTAACTGGAATTACACACTGGCTg                                                                                                                                                                                                    >  1:787400/1‑301 (MQ=255)`  
                                                                                                         `gTATTGGAGCCTGATGGTAAGATAACCGTGTTTGAGCAACCTACGGCTGGGTGGAATAGCGACCCTTGGCTGAGACTCAGCGGTAACTTGGAGGGACGCATGGTGTACATTGGGTTCAACATTAACTTCGTATATGAGTTCTCTAAGTTCCTCATCAAGCAGACTGCCGACGACGGGTCTACCTCCACGGAAGACATTGGGCGCTTACAGTTACGCCGAGCGTGGGTTAACTACGAGAACTCTGGTACGTTTGACATTTATGTTGAGAACCAATCGTCTAACTGGAAGTACACAATGGCTg                                                                                                                                                                                                    >  1:250563/1‑301 (MQ=255)`  
                                                                                                          `gggctcggagatgtgtataagagacagtgtTTGAGCAACCTACGGCTGTGTGGAATAGCGACCCTCGGCTGAGACTCAGCGGTAACTTGGAGGGACGCATGGTGTACATTGGGTTCAACATTAACTTCGTATATGAGTGCTCTAAGTTCCTCATCAAGCAGACTGCCGACGACGGGTCTACCTCCACGGAAGACATTGGGCGCTTACAGTTACGCCGAGCGTGGGTTAACTACGAGAACTCTGGTACATTTGACATTTATGTTGAGAACCAATCGTCTAACTGGAAGTACACAATGGCTgg                                                                                                                                                                                                   <  1:39471/275‑1 (MQ=255)`  
                                                                                                           `aTTGGAGCCTGATGGTAAGATAACCGTGTTTGAGCAACCTACGGCTGGGTGGAATAGCGACCCTTGGCTGAGACTCAGCGGTAACTTGGAGGGACGCATGGTGTACATTGGGTTCAACATTAACTTCGTATATGAGTTCTCTAAGTTCCTAATCAAGCAGACTGCCGACGACGGGTCTACCTCCACGGAAGACATTGGGCGCTTACAGTTACGCCGAGCGTGGGTTAACTACGAGAACTCTGGTACGTTTGACATTTATGTTGAGAACCAATCGTCTAACTGGAAGTACACAATGGCTGGt                                                                                                                                                                                                  >  1:590094/1‑301 (MQ=255)`  
                                                                                                                    `acagggTAAGATAACCGTGTTTGAGCAACCTACGGCTGGGTGGAATAGCGACCCTTGGCTGAGACTCAGCGGTAACTTGGAGGGACGCATGGTGTACATTGGGTTCAACATTAACTTCGTATATGAGTTCTCTAAGTTCCTCATCAAGCAGACTGCCGACGACGGGTCTACCTCCACGGAAGACATTGGGCGCTTACAGTTACGCCGAGCGTGGGTTAACTACGAGAACTCTGGTACGTTTGACATTTATGTTGAGAACCAATCGTCTAACTGGAAGTACACAATGGCTGGTGCCCAATTa                                                                                                                                                                                         <  1:700009/297‑1 (MQ=255)`  
                                                                                                                         `gTAAGATAACCGTGTTTGAGCAACCTACGGCTGGGTGGAATAGCGACCCTTGGCTGAGACTCAGCGGTAACTTGGAGGGACGCATGGTGTACATTGGGTTCAACATTAACTTCGTATATGAGTTCTCTAAGTTCCTCATCAAGCAGACTGCCGACGACGGGTCTACCTCCACGGAAGACGTTGGGCGCTTACAGTTACGCCGAGCGTGGGTTAACTACGAGAACTCTGGTACGTTTGACATTTATGTTGAGAACCAATCATCTAACTGGAAGTACACAATGGCTGGTGCCCGATTAGGctc                                                                                                                                                                                    >  1:316487/1‑301 (MQ=255)`  
                                                                                                                             `gATCACCGTGTTTGAGCAACCTACGGCTGGGTGGAATAGCGACCCTTGGCTGAGACTCAGCGGTAACTTGGATGGACGCATGGTGTACATTGGGTTCAACATTAACTTCGTATATGAGTTCTCTAAGTTCCTCATCAAGCAGACTGCCGACGACGGGTCTACCTCCACGGAAGACATTGGGCGCTTACAGTTACGCCGAGCGTGGGTTAACTACGAGAACTCTGGTACGTTTGACATTTATGTTGAGAACCAATCGTCTAACTGGAAGTACACAATGGCTGGTGCCCGATTAGGCTCTAac                                                                                                                                                                                <  1:805642/301‑1 (MQ=255)`  
                                                                                                                             `gATAACTGTGTTTGAGCAACCTACGGCTGGGTGGAATAGCGACCCTTGGCTGAGACTCAGCGGTAACTTGGAGGGACGCATGGTGTACATTGGGTTCAACATTAACTTCATATATGAGTTCTCTAAGTTCCTCATCAAGCAGACTGCCGACGACGGGTCTACCTCCACGGAAGACATTGGGCGCTTACAGTTACGCCGAGCGTGGGTTAACTACGAGAACTCTGGTACGTTTGACATTTATGTTGAGAACCAATCGTCTAACTGGAAGTACACAATGGcctgtctcttatacacatctccg                                                                                                                                                                                >  1:216543/1‑279 (MQ=255)`  
                                                                                                                             `gATAACCGTGTTTGAGCAACCTACGGCTGGGTGGAATAGCGACCCTTGGCTGAGACTCAGCGGTAACTTGGAGGGACGCATGGTGTACATTGGGTTCAACATTAACTTCGTATATGAGTTCTCTAAGTTCCTCGTCAAGCAGACTGCCGACGACGGGTCTACCTCCACGGAAGACATTGGGCGCTTACAGTTACGCCGAGCGTGGGTTAACTACGAGAACTCTGGTACGTTTGACATTTATGTTGAGAACCAATCGTCTAACTGGAAGTACACAATGGCTGGTGCCCGATTAGGCTCTAac                                                                                                                                                                                <  1:757086/301‑1 (MQ=255)`  
                                                                                                                                 `aCCGTGTTTGAGCAACCTACGGCTGGGTGGGATAGCGACCCTTGGCTGAGACTCAGCGGTAACTTGGATAGACGCATGGTGTACATTGGGTTCAACATTAACTTCGTATATGAGTTCTCTAAGTTCCTCATCAAGCAGACTGCCCACTACGGGTCTACCTCCACGGAAGACATTGGGCGCTTACAGTTACTCCGCACGTGGGTTAACTACGAGAACTCTGGTACGTTTGACATTTATGTTGAGAACCACTCGTCTAACTGGAAGTACACAATGGCTAGTGCCAGTTTATGCGCTacctctt                                                                                                                                                                            >  1:44826/1‑295 (MQ=255)`  
                                                                                                                                 `aCCGTGTTTGAGCAACCTACGGCTGGGTGGAATAGCGACCCTTGGCTGAGACTCAGCGGTAACTTGGAGGGACGCATGGTGTACATTGGGTTCAACATTAACTTCGTATATGAGTTCTCTAAGTTCCTCATCAAGCAGACTGCCGACGACGGGTCTACCTCCACGGAAGACATTGGGCGCTTACAGTTACGCCGAGCGTGGGTTAACTACGAGAACTCTGGTACATTTGACATTTATGTTGAGAACCAATCGTCTAACTGGAAGTACACAATGGCTGGTGCCCGATTAGGCTATAACActc                                                                                                                                                                            <  1:510512/301‑1 (MQ=255)`  
                                                                                                                                   `cGTGTTTGAGCAACCTACGGACGGGTGGAATAGCGACCCTTGGCTGAGACTCAGCGGTAACTTGGAGGGACGCATGGTGTACATTGGGTTCAACATTAACTTCGTATATGAGTTCGCTAAGTTCCTCATCAAGCAGACTGCCGACGACGGGTCTACCTCCACGGAAGACGTTGGGCGCTTACAGTTACGCCGAGCGTGGGTTAACTACGAGAACTCTGGTACGTTTGACATTTATGTTGAGAACCAATCGTCTAACTGGAAGTACACAATGGCTGGTGCCCGATTAGGCTCTAACACTCTg                                                                                                                                                                          <  1:675899/301‑1 (MQ=255)`  
                                                                                                                                         `tGAGCAACCTACGGCTGGGTGGAATAGCGACCCTTGGCTGAGACTCATCGGTAACTTGGAGGGACGCATGGTGTACATTGGGTTCAACATTAACTTCGTATATGAGTTCTCTAAGTTCCTCATCAAGCAGACTGCCGACGACGGGTCTACCTCCACGGAAGCCGTTGGGCGCTTACAGTTACGCCGAGCGTGGGTTAACTACGAGAACTCTGGTACGTTTGACATTTATGTTGAGAACCAATCGTCTAACTGGAAGTACACAATGGCTGGTGCCCGATTAGGCTCTAACACTCTGAGGGCt                                                                                                                                                                    >  1:240004/1‑301 (MQ=255)`  
                                                                                                                                            `cagaCCTACGGCTGGGTGGAATAGCGACCCTTGGCTGAGACTCAGCGGTAACTTGGAGGGACGCATGGTGTACATTGGGTTCAACATTAACTTCGTATATGAGTTCTCTAAGTTCCTCATCAAGCAGACTGCCGACGACGGGTCTACCTCCACGGAAGACGTTGGGCGCTTACAGTTACGCCGAGCGTGGGTTAACTACGAGAACTCTGGTACGTTTGACATTTATGTTGAGAACCAATCGTCTAACTGGAAGTACACAATGGCTGGTGCCCGATTAGGCTCTAACACTCTGAGGGCTggg                                                                                                                                                                 <  1:284595/298‑1 (MQ=255)`  
                                                                                                                                               `aCCTACGGCTGGGTGGAATAGCGACCCTTGGCTGAGACTCAGCGGTAACTTGGATGGACGCATGGTGTACATTGGGTTCAACATTAACTTCGTATATGAGTTCTCTAAGTTCCTCATCAAGCAGACTGCCGACGACGGGTCTACCTCCACGGAAGACGTTGGGCGCTTACAGTTACGCCGAGCGTTGGTTAACTACGAGAACTCAGGTACGTTTGACATTTATGTTGAGAACCAATCGTCTAACTGGAAGTACACAATGGCTGGTGCCCTATTAGGCTCTACCACTCTGGGGGCTGGgaga                                                                                                                                                              >  1:435401/1‑301 (MQ=255)`  
                                                                                                                                               `aCCTACGGCTGGGTGGAATAGCGACCCTTGGCTGAGACTCAGCGGTAACTTGGAGGGACGCATGGTGTACATTGGGTTCAACATTAACTTCGTATATGAGTTCTCTAAGTTCCTCATCAAGCAGACTGCCGACGACGGGTCTACCTCCACGGAAGACGTTGGGCGCTTACAGTTACGCCGAGCGTGGGTTAACTACGAGAACTCTGGTACGTTTGACATTTATGTTGAGAACCAATCGTCTAACTGGAAGTACACAATGGCTGGTGCCCGATTAGGCTCTAACACTCTGAGGGCTGGgagc                                                                                                                                                              >  1:468152/1‑300 (MQ=255)`  
                                                                                                                                               `aCCTACGGCTGGGTGGAATAGCGACCCTTGGCTGAGACTCAGCGGTAACTTGGAGGGACGCATGGTGTACATTGGGTTCAACATTAACTTCGTATATGAGTTCTCTAAGTTCCTCATCAAGCAGACTGCCGACGACGGGTCTACCTCCACGGAAGACGTTGGGAGCTTACAGTTACGCCGAGCGTGGGTTAACTACGAGAACTCTGGTACGTTTGACATTTATGTTGAGAACCAATCGTCTAACTGGAAGTACACAATGGCTGGTGCCCGAttctgtctcttatacacatctccgagcccc                                                                                                                                                              >  1:205242/1‑273 (MQ=255)`  
                                                                                                                                               `aCCTACGGCTGGGTGGAATAGCGACCCTTGGCTGAGACTCAGCGGTAACTTGGAGGGACGCATGGTGTACATTGGGTTCAACATTAACTTCGTATATGAGTTCTCTAAGTTCCTCATCAAGCAGACTGCCGACGACGGGTCTACCTCCACGGAAGACATTGGGCGCTTACAGTTACGCCTAGCGTGGTTTAACTACGAGAACTCTGGTACGTTTGACATTTATGTTGAGAACCAATCGTCTAACTGGAAGTACACAATGGCTGGTGCCCGATTAGGCTCTAACACTCTGAGGGCTgtctct                                                                                                                                                              >  1:603259/1‑296 (MQ=255)`  
                                                                                                                                               `aCCTACGGCTGGGTGGAATAGCGACCATTGGCTGAGACTCAGCGGTAACTTGGAGGGACGCATGGTGTACATTGGGTTCAACATTAACTTCGTATATGAGTTCTCTAAGTTCCTCATCAAGCAGACTGACGACGACGGGTCTACCTCCACGGAAGACATTGGGCGCTTACAGTTACGCCGAGCGTGGGTTAACTACGAGAACTCTGGTACGTTTGACATTTATGTTGAGAACCAATCGTCTAACTGGAAGTACACAATGGCTGGTGCCCGATTAGGCTCTAACACTCTGAGGGCTGGgaga                                                                                                                                                              >  1:685316/1‑301 (MQ=255)`  
                                                                                                                                                   `aCGGCTGGGTGGAATAGCGACCCTTGGCTGAGACTCAGCGGTAACTTGGAGGGACGCATGGTGTACATTGGGTTCAACATTAACTTCGTATATGAGTTCTCTAAGTTCTTCATCAAGCAGACTGCCGACGACGGGTCTACCTCCACGGAAGACGTTGGGCGCTTACAGTTACGCCGAGCGTGGGTTAACTACGAGAACTCTGGTACGTTTGACATTTATGTTGAGAACCAATCGTCTAACTGGAAGTACACAATGGCTGGTGCCCGATTAGGCTCTAacacctgtctcttatacacatctc                                                                                                                                                          >  1:545432/1‑281 (MQ=255)`  
                                                                                                                                                     `ggCTGGGTGGAATAGCGAACCTTGGCTGAGACGCAGCGGTAACTTGGAGGGACGCATGGTGTACATTGGGTTCAACATTAACTTCGTATATGAGTTCTCTAAGTTCCTCATCAAGCAGACTGCCGACGACGGGTCTACCTCAACGGAAGACATTGGGCGATTACAGTTACGCCGAGCGTGGGTTAACTACGAGAACTCTGGTACGTTTGACATTTATGTTGAGAACCAATCGTCTAACTGGAAGTACACAATGGCTGGTGCCCGATTAGGCTCTAACACTATGAGGGCTGGGAGACTGAAc                                                                                                                                                        <  1:421851/301‑1 (MQ=255)`  
                                                                                                                                                         `gggTGGAATAGCGACCCTTGGCTGAGACTCAGCGGTAACTTGGAGGGACGCATGGTGTACATTGGGTTCAACATTAACTTCGTATATGAGTTCTCTAAGTTCCTCATCAAGCAGACTGCCGACGACGGGTCTACCTCCACGGAAGACATTGGGCGCTTACAGTTACGCCGAGCGTGGGTTAACTACGAGAACTCTGGTACGTTTGACATTTATGTTGAGAACCAATCGTCTAACTGGAAGTACACAATGGCTGGTGCCCGATTAGGCTCTAACACTCTGAGTGCTGGGAGACTGCACTTAg                                                                                                                                                    >  1:45410/1‑301 (MQ=255)`  
                                                                                                                                                           `gTGGAATAGCGACCCTTGGCTTAGACTCAGCGGTAACTTGGTGGGACGCATGGTGTACATTGGGTTCAACATTAACTTCGTATATGAGTTCTCTAAGTTCCTCATCAAGCAGACTGCCGACGACGGGTCTACCTCCACTGAAGACATTGGGCGCTTACAGTTACGCCGAGCGTGGGTTAACTACGAGAACTCTGGTACGTTTGACATTTATGTTGAGAACCAATCGTCTAACTGGAAGTACACAATGGCTGGTGCCCGATTAGGCTCTAACACTCTGAGGGCTGGGAGACTGAACTTAggg                                                                                                                                                  <  1:523137/301‑1 (MQ=255)`  
                                                                                                                                                           `gTGGAATAGCGACCCTTGGCTGAGACTCAGCGGTAACTTGGAGGGACGCATGGTGTACATTGGGTTCAACATTAACTTCGTATATGAGTTCTCTAAGTTCCTCATCAAGCAGACTGCCGACGACGGGTCTACCTCCACGGAAGACGTTGGGCGCTTACAGTTACGCCGAGCGTGGGTTAACTACGAGAACTCTGGTACGTTTGACATTTATGTTGAGAACCAATCGTCTAACTGGAAGTACACAATGGCTGGTGCCCGATTAGGCTCTAacacctgtctcttatacacatctccgagccca                                                                                                                                                  >  1:61401/1‑273 (MQ=255)`  
                                                                                                                                                           `gTGGAATAGCGACCCTTGGCTGAGACTCAGCGGTAACTTGGAGGGACGCATGGTGTACATTGGGTTCAACATTAACTTCGTATATGAGTTCTCTAAGTTCCTCATCAAGCAGACTGCCGACGACGGGTCTACCTCCACGGAAGACATTTGGCGCTTACAGTTACGCCGAGCGTTGGTTAACTACGAGAACTCTGGTACGTTTGACATTTATGTTGAGAACCAATCGTCTAACTGGAAGTACACAATGGCTGGTGCCCGATTAGGCTCTAACACTCTGAGGGCTGGGAGACTGAACTTAggg                                                                                                                                                  >  1:162474/1‑301 (MQ=255)`  
                                                                                                                                                             `tgAATAGCGACCCTTGGCTGAGACTCAGCGGTAACTTGGAGGGACGCATGGTGTACATTGGGTTCAACATTAACTTCGTATATGAGTTCTCTAAGTTCCTCATCAAGCAGACTGCCGACGACGGGTCTACCTCCACGGAAGACATTGGGCGCTTACAGTTACGCCGAGCGTGGGTTAACTACTAGAACTCTGGTACGTTTGACATTTATGTTGAGAACCAATCGTCTAACTGGAAGTACACAATGGCTGGTGCCCGATTAGGCTCTAACACTCTGAGGGCTGGGAGACTGAACTTAGGGAc                                                                                                                                                <  1:237250/300‑1 (MQ=255)`  
                                                                                                                                                                `aTAGCGACCATTGGCTGAGACTCAGAGGTAACTTGGAGGGACGCATGGTGTACATTGGGTTACACATTAACTTCGTATATGAGTTCTCTAAGTTCCTCATCAAGCAGACTGCCGACGACGGGTCTACCTCCACGGAAGACATTGGGCGCTTACAGTTACGCCGAGCGTGGGTTAACTACGAGCACTCTGGTACGTTTGACATTTATGTTGAGAACCAATCGTCTAACTGGAAGTACACAATGGCTGGTGCCCGATTAGGCTCTAACACTCTGAGGGCGGGGAGACTGAAcctttctcttaa                                                                                                                                             >  1:66608/1‑290 (MQ=255)`  
                                                                                                                                                                  `acagACCCTTGGCTGAGACTCAGCGGTAACTTGGAGGGACGCATGGTGTACATTGGGTTCAACATTAACTTCGTATATGAGTTCTCTAAGTTCCTCATCAAGCAGACTGCCGACGACGGGTCTACCTCCACGGAAGACGTTGGGCGCTTACAGTTACGCCGAGCGTGGGTTAACTACGAGAACTCTGGTACGTTTGACATTTATGTTGAGAACCAATCGTCTAACTGGAAGTACACAATGGCTGGTGCCCGATTAGGCTCTAACACTCTGAGGGCTGGGAGACTGAACTTAGGGACCGGAc                                                                                                                                           <  1:579181/298‑1 (MQ=255)`  
                                                                                                                                                                  `aGCGACCCTTGGCTGAGACTCAGCGGTAACTTGGAGGGACGCATGGTGTACATTGGGTTCAACATTAACTTCGTATATGAGTTCTCTAAGTTCCTCATCAAGCAGACTGCCGACGACGGGTCTACCTCCACGGAAGACATTGGGCGCTTACAGTTACGCCGAGCGTGGGTTAACTACGAGAACTCTGGTACGTTTGACATTTATGTTGAGAACCAATCGTCTAACTGGAAGTACACAATGGCTGGTGCCCGATTAGGCTCTAACACTCTGAGGGCTGGGAGACTGAACTTAGGGACCGGAc                                                                                                                                           <  1:475936/301‑1 (MQ=255)`  
                                                                                                                                                                  `aGCGACCATTGGATGAGACTCAGCGGTAACTTGGAGGGACGCATGGTGTACATTGGGTTCAACATTAACTTCGTATATGAGTTCTCTAAGTTCCTCATCAAGCAGACTGCCGACGACGGGTCTACCTCCACGGAAGACGTTGGGCGCTTACAGTTACGCCGAGCGTGGGTTAACTACGAGAACTCTGGTACGTTTGACATTTATGTTGAGAACCAATCGTCTAACTGGAAGTACACAATGGCTGGTGCCCGATTAGGCTCTAACACTCTGAGGGCTGGGAGACTGAACTTAGGGACCGGAc                                                                                                                                           <  1:820287/301‑1 (MQ=255)`  
                                                                                                                                                                       `cccTTGGCTGAGACTCAGCGGTAACTTGGAGGGACGCATGGTGTACATTGGGTTCAACATTAACTTCGTATATGAGTTATCTAAGTTCCTCATCAAGCAGACTGCCGACGACGGGTCTACCTCCACGGAAGACATTGGGCGCTTACAGTTACGCCGAGCGTGGGTTAACTACGAGAACTCTGGTACGTTTGACATTTATGTTGAGAACCAATCGTCTAACTGGAAGTACACAATGGCTGGTGCCCGATTAGGCTCTAACACTCTGAGGGCTgtctcttatacacatctccgagcccacgag                                                                                                                                      >  1:599282/1‑272 (MQ=255)`  
                                                                                                                                                                            `ggCTGAGACTCAGCTGTAACGTGGATGGACGCATGGTGTACATTGGGTTCAACATTAAATTCGTATATGAGTTCTCTAAGTTCCTCATCAAGCAGACTGCCGACGACTGGTCTACCTCCACGGAAGACATTTGGCGCTTACAGTTACGCCGAGCGTGGGTTAACTACGAGAACTCTGGTACGTTTGACATTTATGTTGAGAACCAATCGTCTAACTGGAAGTACACAATGGCTGGTGCCCGATTAGGCTCTAACACTCTGAGGGCTGGGAGACTGAACTTAGGGACCGGACAATATAGAtt                                                                                                                                 <  1:743320/301‑1 (MQ=255)`  
                                                                                                                                                                            `ggCTGAGACTCAGCGGTAACTTGGAGTGACGCATGGTGTACATTGGGTTCAACATTAACTTCGTATATGAGTTCTCTAAGTTCCTCATCAAGCAGACTGCCGACGACGGGTCTACCTCCACGGAAGACATTGTGCGCTTACAGTTACGCCGAGCGTGGGTTAACTACGAGAACTCTGGTACGTTTGACATTTATGTTGAGAACCAATCGTCTAACTGGAAGTACACAATGGCTGGTGCCCGATTAGGCTCTAACACTCTGAGGGCTGGGAGACTGAACTTAGGGACCGGACAATATCGAtt                                                                                                                                 <  1:443130/301‑1 (MQ=255)`  
                                                                                                                                                                            `ggCTGAGACTCAGCGGTAACTTGGAGGGACTCATGGTGTACATTGGGTTCAACATTAACTTCGTATATGAGTTCTCTAAGTTCCTCATCAAGCAGACTGCCGACGACGGGTCTACCTCCACGGAAGACATTGGGCGCTTACAGTTACGCCGAGCGTGGGTTAACTACGAGAACTCTGGTACGTTTGACATTTATGTTGAGAACCAATCGTCTAACTGGAAGTACACAATGGCTGGTGCCCGATTAGGCTCTAACACTCTGAGGGCTTGGAGACTGAACTTAgggctgtctcttatacacat                                                                                                                                 >  1:738781/1‑284 (MQ=255)`  
                                                                                                                                                                            `ggCTGAGACTCAGCGGTAACTTGGAGGGACGCATGGTGTACATTGGGTTCAACATTAACTTCGTATATGAGTTCTCTAAGTTCCTCATCAAGCAGACTGCCGACGACGGGTCTACCTCCACGGAAGACATTGGGCGCTTACAGTTACGCCGAGCGTGGGTTAACTACGAGAACTATGGTACATTTGACATTTATGTTGAGAACCAATCGTCTAACTGGAAGTACACAATGGCTGGTGCCCGATTAGGCTCTAACACTCTGAGGGCTGGGAGACTGAACTTAGGGACCGGACAATATCGAtt                                                                                                                                 >  1:29037/1‑301 (MQ=255)`  
                                                                                                                                                                             `gcggagaCTCAGCGGTAACTGGGAGGGACGCATGGTGTACATTGGGTTCAACATGAACTTCGTATATGAGTTCTCTAAGTTCCTCATCAAGCAGACTGCCGACGACTGGGCTACCTCCACGGAAGACATTGGGCGCTTACAGGTACGCCGAGCGTGGGTTAACTACGAGAACTCTGGTACGTTTGACATTTATGTTGAGAACCAATCGTCTAACTGGAAGTACACAATGGCTGGTGCACGATTAGGCTCTAACACTCTGAGGGCTGGGAGACTGAACTTAGGGACCGGACAATATCGATTc                                                                                                                                <  1:259510/298‑1 (MQ=255)`  
                                                                                                                                                                                 `agaCTCAGCGGTAACTTGGAGGGACGCATGGTGTACATTGGGTTCAACATTAACTTCGTATATGAGTTCTCTAAGTTCCTCATCAAGCAGACTGCCGACGACGGGTCTACCTCCACGGAAGACATTGGGCGCTTACAGTTACGCCGAGCGTGGGTTAACTACGAGAACTCTGGTACGTTTGACATTTATGTTGAGAACCAATCGTCTAACTGGAAGTACACAATGGCTGGTGCCCGATTAGGCTCTAACACTCTGAGGGCTGGGAGACTGAACTTAGGGACCGGACAATATCGATTCCCtg                                                                                                                            <  1:720984/301‑1 (MQ=255)`  
                                                                                                                                                                                         `cGGTAACTTGGAGGGACGCATGGTGTACATTGGGTTCAACATTAACTTCGTATATGAGTTCTCTAAGTTCCTCATCAAGCAGACTGCCGACGACGGGTCTACCTCCACGGAAGACATTGGGCGCTTACAGTTACGCCGAGCGTGGGTTAACTACGAGAACTCTGGTACGTTTGACATTTATGTTGAGAACCAATCGTCTAACTGGAAGTACACAATGGCTGGTGCCCGATTAGGCTCTAACACTCTGAGGGCTGGGAGACTGAACTTAGGGACCAGACAATATCGATTCCCTGtggttggt                                                                                                                    <  1:401390/301‑1 (MQ=255)`  
                                                                                                                                                                                                   `gAGGGACGCATGGTGTACATTGGGTTCAACATTAACTTCGTATATGAGTTCTCTAAGTTCCTCATCAAGCAGACTGCCGACGACGGGTCTACCTCCACGGAAGACATTGGGCGCTTACAGTTACGCCGAGCGTGGGTTAACTACGAGAACTCTGGTACGTTTGACATTTATGTTTAGAACCAATCGTCTAACTGGAAGTACACAATGGCTGGTGCCCGATTAGGCTCTAACACTCTGAGGGCTGGGATACTGAACTTAGGGACCGGACAATATCGATTCCCTGTTGTTGGTAACGCCAAGt                                                                                                          >  1:264697/1‑301 (MQ=255)`  
                                                                                                                                                                                                          `ggagatgtgtataagagacagaCATTAACTTCGTATATGAGTTCTCTAAGTTCCTCATCAAGCAGACTGCCGACGACGGGTCTACCTCCACGGAAGACGTTGGGCGCTTACAGTTACGCCGAGCGTGGGTTAACTACGAGAACTCTGGTACGTTTGACATTTATGTTGAGAACCAATCGTCTAACTGGAAGTACACAATGGCTGGTGCCCGATTAGGCTCTAACACTCTGAGGGCTGGGAGACTGAACTTAGGGACCGGACAATATCGATTCCCTGTGGTTGGTAACGCCAAGTTCAacac                                                                                                   <  1:152698/280‑1 (MQ=255)`  
                                                                                                                                                                                                          `gacaggTGTACATTGGGTTCAACATTAACTTCGTATATGAGTTCTATAAGTTCCTCATCAAGCAGACTGCCGACGACGGGTCTACCTCCACGGAAGACATTGGGCGCTTACAGTTACGCCGAGCGTGGGTTAACTACGAGAACTCTGGTACGTTTGACATTTATGTTGAGAACCAATTGTCTAACTGGAAGTACACAATGGCTGGTGTCCGATTAGGCTCTAACACTCTGAGGGCTGGGAGACTGAACTTAGGGACCGGACAATATCGATTCCCTGTGGTTGGTAACGCCAAGTTCAacac                                                                                                   <  1:535268/297‑1 (MQ=255)`  
                                                                                                                                                                                                          `gCATGGTGTACATTGGGTTTAACATTAACTTCGTATATGAGTTCTCTAAGTTCCTCATCAAGCAGACTGCCGACGACGGGTCTACCTCCACGGAAGACGTTGGGCGCTTACAGTTACGCCGAGCGTGGGTTAACTACGAGAACTCTGGTACGTTTGACATTTATGTTGAGAACCAATCGTCTAACTAGAAGTACACAATGGCTGGTGCCCGATTAGGCTCTAACACTCTGAGGGCTGGGAGACTGAACTTAGGGACCGGACAATATCGATTCCCTGTGGTTGGTAACGCCAAGTTCAacac                                                                                                   <  1:358120/301‑1 (MQ=255)`  
                                                                                                                                                                                                          `gCATGGTGTACATTGGGTTCAACATTAACTTCGTATATGAGTTCTCTAAGTTCCTCATCAAGCAGACTTCCGACTACGGGTCTACCTCCACGGAAGACATTGGGCGCTTACAGTTACGCCGAGCGTGGGTTAACTACGAGAACTCTGGTACGTTTGACATTTATGTTGAGAACCAATCGTCTAACTGGAAGTACACAATGGCTGGTGCCCGATTAGGCTCTAACACTCTTAGGGCTGGGAGACTGAACTTAGGGACCGGACAATATCGATTCCCTGTGGTTGGTAACGCCAAGTTCAacac                                                                                                   <  1:100147/301‑1 (MQ=255)`  
                                                                                                                                                                                                          `gCATGGTGTACATTGGGTTCAACATTAACTTCGTATATGAGTTCTCTAAGTTCCTCATCAAGCAGACTGCCGTCGACGGGTCTACCTCCACGGAAGACGTTGGGCGCTTACAGTTACGCCGAGCGTGGGTTAACTACGAGAACTCTGGTACGTTTGACATTTATGTTGAGAACCAATCGTCTAACTGGAAGTACACAATGGCTGGTGCCCGATTAGGCTCTAACACTCTGAGGGCTTGGAGACTGAACTTAGGGACCGGACAATATCGATTCCCTGTGGTTGGTAACGCCAAGTTCAacac                                                                                                   <  1:56576/301‑1 (MQ=255)`  
                                                                                                                                                                                                            `agatgtgtataagagacagaCATTAACTTCGTATATGAGTTCTCTAAGTTCCTCATCAAGCAGACTGCCGACGACGGGTCTACCTCCACGGAAGACGTTGGGCGCTTACAGTTACGCCGAGCGTGGGTTAACTACGAGAACTCTGGTACGTTTGACATTTATGTTGAGAACCAATCGTCTAACTGGAAGTACACAATGGCTGGTGCCCGATTAGGCTCTAACACTCTGAGGGCTGGGAGACTGAACTTAGGGACCGGACAATATCGATTCCCTGTGGTTGGTAACGCCAAGTTCAACACTg                                                                                                 <  1:714433/282‑1 (MQ=255)`  
                                                                                                                                                                                                             `tGGTGTACATTGGGTTCAACATTAACTTCGTATATGAGTTCTCTAAGTTCCTCATCAAGCAGACTGCCGACGACGGGTCTACCTCCACGGAAGACATTGGGCGCTTACAGTTACGCCGAGCGTGGGTTAACTACGAGAACTCTGGTACGTTTGACATTTATGTTGAGAACCAATCGTCTAACTGGAAGTACACAATGGCTGGTGCCCGATTAGGCTCTAACACTATGAGGGATGGGAGACTGAACTTAGGGACCGGAAAATATCGATTCCATGTGGTTGGTAACGCTAAGTACAACACTGt                                                                                                >  1:553787/1‑301 (MQ=255)`  
                                                                                                                                                                                                               `gtgtACATTGGGTTCAACATTAACTTCGTATATGAGTTCTCTAAGTTCCTCATCAAGCAGACTGCCGACGACGGGTATACCTCCACGGAAGACATTGGGCGCTTACAGTTACGCCGAGCGTGGGTTAACTACGAGAACTCTGGTACGTTTGACATTTATGTTGAGAACCAATCGTCTAACTGGAAGTACACAATGGCTGGTGCCCGATTAGGCTCTAACACTCTGAGGGCTGGGAGACTGAACTTAGGGACCGGACAATATCGATTCCCTGTGGTTGGTAACGCCAAGTTCAacacctgtc                                                                                              >  1:124175/1‑296 (MQ=255)`  
                                                                                                                                                                                                               `gtgtACATTGGGTTAAACATTAACTTCGTATATGAGTTCTCTAAGTTCCTCATCAAGCAGACTGCCGACGACGGGTCTACCTCCACGGAAGACATTGGGCGCTTACAGTTACGCCGAGCGTGGGTTAACTACGAGAACTCTGGTACGTTTGACATTTATGTTGAGAACCAATCGTCTAACTGGAAGTACACAATGGCTGGTGCCCGATTAGGCTCTAACACTCTGAGGGCTGGGAGACTGAACTTAGGGACCGGACAATATCGATTCCCTGTGGTTGGTAACGCCAAGTTCAACACTGtat                                                                                              <  1:300301/301‑1 (MQ=255)`  
                                                                                                                                                                                                               `gggctcggagatgtgtctaagagacaggTATATGAGTTCTCTAAGTTCCTCATCAAGCAGACTGCCGACGACGGGTCTACCTCAACGGAAGACGTTGGGCGCTTACAGTTACGCCGAGCGTGGGTTAACTACGAGAACTCTGGTACGTTTGACATTTATGTTGAGAACCAATCGTCTAACTGGAAGTACACAATGGCTGGTGCCCGATTAGGCTATAACACTCTGAGGGCTGGGAGACTGAACTTAGGGACCGGACAATATCGATTCCCTGTGGTTGGTAACGCCAAGTTCAACACTGtat                                                                                              <  1:812283/274‑1 (MQ=255)`  
                                                                                                                                                                                                                 `ttACATTTGGTTCAACATTAACTTCGTATATGAGTTCTCTAAGTTCCTCATCAAGCAGACTGCCGACGACGGGTCTACCTCCACGGAAGACATTGGGCGATTACAGTTACGCCGAGCGTGGGTTAACTACGAGAACTCTGGTACGTTTGACATTTATGTTGAGAACCAATCGTCTAACTGGAAGTACACAATGGCTGGTGCCCGATTAGGCTCTAACACTCTGAGGGCTGGGAGACTGAACTTAGGGACCGGACAATATCGATTCCCTGTGGTTGGTAACGCCAAGTTCAACACTGTATAc                                                                                            <  1:762447/300‑1 (MQ=255)`  
                                                                                                                                                                                                                 `gtACATTGGGTTCAACATTAACTTCGTATATGAGTTCTCTAAGTTCCTCATCAAGCAGACTGCCGACGACGGGTCTACCTCCACGTAAGACATTGTGCGCTTACAGTTACGCCGAGCGTGGGTTAACTACGAGAACTCTGGTACGTTTGACATTTATGTTGAGAACCAATCGTCTAACTGGAAGTACACAATGGCTGGTGCCCGATTAGGCTCTAACACTCTGAGGGCTGGGAGACTGAACTTAGGGACCGGACAATATCGATTCCCTGTGGTTGGTAACGCCAAGTTCAACACTGTATAc                                                                                            <  1:411538/301‑1 (MQ=255)`  
                                                                                                                                                                                                                  `cggagatgtgtataagagacagtCGTATATGAGTTCTCTAAGTTCCTCATCAAGCAGACTGCCGACGACGGGTCTACCTCCACGGAAGACATTGGGCGCTTATAGTTACGCCGAGCGTGGGTTAACTACGAGAACTCTGGTACGTTTGACATTTATGTTGAGAACCAATCGTCTAACTGGAAGTACACAATGGCTGGTGCCCGATTAGGCTCTAACACTCTGAGGGCTGGGAGACTGAACTTAGGAACCGGACAATATCGATTCCCTGTGGTTGGTAACGCCAAGTTCAACACTGTATACa                                                                                           <  1:567830/279‑1 (MQ=255)`  
                                                                                                                                                                                                                  `agagacaggTTCAACATTAACTTCGTATATGAGTTCTCTAAGTTCCTCATCAAGCAGACTGCCGACGACGGGTCTACCTCCACTGAAGACATTGGGCGCTTACAGTTACGCCGAGCGTGGGTTAACTACGAGAACTCTGGTACGTTTGACATTTATGTTGAGAACCAATCGTCTAACTGGAAGTACACAATGGCTTGTGCCCGATTAGGCTCTAACACTCTGAGGGCTTGGAGACTGAACTTAGGGACCGGACAATATCGATTCCCTGTGGTTGGTAACGCCAAGTTCAACACTGTATACa                                                                                           <  1:710061/294‑1 (MQ=255)`  
                                                                                                                                                                                                                    `cATTTGGTTCAACATTAACTTCGTATATGAGTTCTCTAAGTTCCTCATCAAGCAGACTGCCGACGACGGGTCTACCTCCACGGAAGACGTTGGGCGCTTACAGTTACGCCGAGCGTGGGTTAACTACGAGAACTCTGGTACGTTTGACATTTATGTTGAGAACCAATCGTCTAACTGGAAGTACACAATGGCTGGTGCCCGATTAGGCTCTAACACTCTGAGGGCTGGGAGACTGAACTTAGGGACCGGACAATATCGATTCCCTGTGGTTGGTAACGCCAAGTTCAACACTGTATACATc                                                                                         <  1:10700/301‑1 (MQ=255)`  
                                                                                                                                                                                                                    `cATTGGGTTCAACATTATCTTCGTATATGAGTTCTCTAAGTTCCTCATCAAGCAGACTGCCTACGACGGGTCTACCTCCACGGAAGACATTGGGCGCTTACAGTTACGCCGAGCGTGGGTTAACTACGAGAACTCTGGTACGTTTGACATTTATGTTGAGAACCAATCGTCTAACTGGAAGTACACAATGGCTGGTGCCCGATTAGGCTCTAACACTCTGAGGGCTGGGAGACTGAACTTAGAGACCGGACAATATCGATTCCCTGTGGTTGGTAACGCCAAGTTCAACACTGTATACATc                                                                                         <  1:75472/301‑1 (MQ=255)`  
                                                                                                                                                                                                                    `cATTGGGTTCAACATTAACTTCGTATATGAGTTCTCTAAGTTCCTCATCAAGCAGACTGCCGACGACGGGTCTACCTCCACGGAAGACGTTGGGCGCTTACAGTTACGCCGAGCGTGGGTTAACTACGAGAACTCTGGTACGTTTGACATTTATGTTGAGAACCAATCGTCTAACTGGAAGTACACAATGGCTGGTGCCCGATTAGGCTCTAACACTCTGAGGGCTTGGAGACTGAACTTAGGGACCGGACAATATCGATTCCCTGTGGTTGGTAACGCCAAGTTCAACACTGTATACATc                                                                                         <  1:501066/301‑1 (MQ=255)`  
                                                                                                                                                                                                                    `cATTGGGTTCAACATTAACTTCGTATATGAGTTCTATAAGTTCCTCATCAAGCAGACTGCCGACGACGGGTCTACCTCCACGGAAGACGTTGGGCGCTTACAGTTACGCCGAGCGTGGGTTAACTACGAGAACTCTGGTACGTTTGACATTTATGTTGAGAACCAATCGTCTAACTGGAAGTACACAATGGCTGGTGCCCGATTAGGCTCTAACACTCTGAGGGCTGGGAGACTGAACTTAGGGACGGGACAATATCGATTCCCTGTGGTTGGTAACGCCAAGTTCAACACTGTATACATc                                                                                         <  1:637732/301‑1 (MQ=255)`  
                                                                                                                                                                                                                    `cATTGGGGTCAACATTAACTTCGTATATGAGTTCTCTAAGTTCCTCATCAAGCAGACTGCCGACGACGGGTCTACCTCCACGGAAGACGTTGGGCGCTTACAGTTACGCCGAGCGTGGGTTAACTACGAGAACTCTGGTACGTTTGACATTTATGTTGAGAACCAATCGTCTAACTGGAAGTACACAATGGCTGGTGCCCGATTAGGCTCTAACACTCTGAGGGCTGGGAGACTGAACTTAGGGACCGGACAATATCGATTCCCTGTGGTTGGTAACGCCAAGTTCAACACTGTATACATc                                                                                         <  1:342987/301‑1 (MQ=255)`  
                                                                                                                                                                                                                    `aaTTGGGTTCAACATTAACTTCTTATATGAGTTCTCTAAGTTCCTCATCAAGCAGACTGCCGACGACTGGTCTACCTCCACGGAAGACATTGGTCGCTTACAGTTACGCCGAGCGTGGGTTAACTACGAGAACTCTGGTACGTTTGACATTTATGTTGAGAACCAATCGTCTAACTGGAAGTACACAATGGCTGGTGCCCGATTGGGCTCTAACACTCTGAGGGCTGGGAGACTGAACTTAGGGACCGGACAATATCGATTCCCTGTGGTTGGTAACGCCAAGTTCAACACTGTATACATc                                                                                         <  1:295183/300‑1 (MQ=255)`  
                                                                                                                                                                                                                        `gggTTCAACATTAACTTCGTATATGAGTTCTCTAAGTTCCTCATCAAGCAGACTGCCGACGACGGGTCTACCTACACGGAAGACATTGGGCGCTTACAGTTACGCCGAGCGTGGGTTAACTACGAGAACTCTGGTACGTTTGACATTTATGTTGAGAACCAATCGTCTAACTGGAAGTACACAATGGCTGGTGCCCGATTAGGCTCTAACACTCTGAGGGCTGGGAGACTGAACTTAGGGACCGGACAATATCGATTCCCTGTGGTTGGTAACGCCAAGTTCAacctgtctcttatacaca                                                                                     >  1:800516/1‑285 (MQ=255)`  
                                                                                                                                                                                                                              `aaCATTAACTTCGTATATGAGTTCTCTAAGTTCCTCATCAAGCAGACTGCCGACGACGGGTCTACCTCCACGGAAGACATTGGGCGCTTACAGTTACGCCGATCGTGGGTTAACTACGAGAACTCTGGTACGTTTGACATTTATGTTGAGAACCAATCGTCTAACTGGAAGTACACAATGGCTGGTGCCCGATTAGGCTCTAACACTCTGAGGGCTGGGAGACTGAACTTAGGGACCGGACAATATCGATTCCCTGTGGTTGGTAACGCCAAGTTCAACACTGTATACATCTTGTCAGATg                                                                               <  1:736568/301‑1 (MQ=255)`  
                                                                                                                                                                                                                                `cATTAACTTCGTATATGAGTTCTCTAAGTTCCTCATCAAGCAGACTGCCAACGACGGGTCTACCTCCACGGAAGACATTGGGCGCTTACAGTTACGCCGAGCGTGGGTTAACTACGAGAACTCTGGTACGTTTGACATTTATGTTGAGAACCAATCGTCTAACTGGAAGTACACAATGGCTGGTGCCCGATTAGGCTCTAACACTCTGAGGGCTGGGAGACTGAACTTAGGGACCGGACAATATCGATTCCCTGTGGTTGGTAACGCCAAGTTCAACACTGTATACATCTTGTCAGATgag                                                                             >  1:460618/1‑301 (MQ=255)`  
                                                                                                                                                                                                                                        `aggTATATGAGTTCTCTAAGTTCCTCATCAAGCAGACTGCCGACGACGGGTCTACCTCCACGGAAGACGTTGGGCGCTTACAGTTACGCCGAGCGTGGGTTAACTACGAGAACTCTGGTACGTTTGACATTTATGTTGAGAACCAATCGTCTAACTGGAAGTACACAATGGCTGGTGCCCGATTAGGCTCTAACACTCTGAGGGCTGGGAGACTGAACTTAGGGACCGGACAATATCGATTCCCTGTGGTTGGTAACGCCAAGTTCAACACTGTATACATCTTGTCAGATGAGACTAcccc                                                                     <  1:142253/299‑1 (MQ=255)`  
                                                                                                                                                                                                                                          `gTATATGAGTTCTCTAAGTTCCTAATCAAGCAGACTGCCGACGACGGGTCTACCTCCACGGAAGACATTGGGCGCTTACAGTTACGCCGAGCGTGGGTTAACTACGAGAACTCTGGTACGTTTGACATTTATGTTGAGAACCAATCGTCTAACTGGAAGTACACAATGGCTGGTGCCCGATTAGGCTCTAACACTCTGAGGGCTGGGAGACTGAACTTAGGGACCGGACAATATCGATTCCCTGTGGTTGGTAACGCCAAGTTCAACACTGTATACATCTTGTcctgtctcttatacacat                                                                   >  1:57414/1‑284 (MQ=255)`  
                                                                                                                                                                                                                                          `gTATATGAGTTCTCTAAGTTCCTAATCAAGCAGACTGCCGACGACGGATCTACCTCCACGGAAGACATTGGGCGCTTACAGTTACGCCGAGCGTGGGTTAACTACGAGAACTCTGGTACGTTTGACATTTATGTTGAGAACCAATCGTCTAACTGGAAGTACACAATGGCTGGTGCCCGATTAGGCTCTAACACTCTGAGGGCTGGGAGACTGAACTTAGGGACCGGACAATATCGATTCCCTGTGGTTGGTAACGCCAAGTTCAACACTGTATACATCCTGTctcttatacacatatccg                                                                   >  1:186803/1‑284 (MQ=255)`  
                                                                                                                                                                                                                                               `caggTTCTCTAAGTGCCGCAGCAAGCAGACGGCCGACGACGGGTCTACCTCCACGGAAGACGTTGGGCGCTTACAGTTACGCCGAGCGTGGGTTAACTACGAGAACTCTGGTACGTTTGACATTTATGTTGAGAACCAATCGTCTAACTGGAAGTACACAATGGCTGGTGCCCGATTAGGCTCTAACACTCTGAGGGCTGGGAGACTGAACTTAGGGACCGGACAATATCGATTCCCTGTGGTTGGTAACGCCAAGTTCAACACTGTATACATCTTGTCAGATGAGACTACCCCTCTGAAc                                                              <  1:244967/298‑1 (MQ=255)`  
                                                                                                                                                                                                                                               `agagacagctAAGGTCCTCATCAAGCAGACGGCCGACGACGGGTCTACCTCCACGGAAGACGTTGGGCGCTTACAGTTACGCCGAGCGTGGGTTAACTACGAGAACTCTGGTACGTTTGACATTTATGTTGAGAACCAATCGTCTAACTGGAAGTACACAATGGCTGGTGCCCGATTAGGCTCTAACACTCTGAGGGCTGGGAGACTGAACTTAGGGACAGGACAATATCGATTCCCTGTGGTTGGTAACGCCAAGTTCAACACTGTATACATCTTGTCAGATGAGACTACCCCTCTGAAc                                                              <  1:484684/293‑1 (MQ=255)`  
                                                                                                                                                                                                                                                 `aGTTCTCTAAGTTCCTCATCAAGCAGACTGCCGACGACGGGTCTACCTCCACGGAAGACGTTGGGCGCTTACAGTTACGCCGAGCGTGGGTTAACTACGAGAACTCTGGTACGTTTGACATTTATGTTGAGAACCAATCGTCTAACTGGAAGTACACAATGGCTGGTGCCCGATTAGGCTCTAACACTCTGAGGGCTGGGAGACTGAACTTAGTGACCGGACAATATCGATTCCCTGTGGTTGGTAACGCCAAGTTCAACACTGTATACATCTTGTCAGATGAGACTACCCCTCTGAAcat                                                            <  1:583131/301‑1 (MQ=255)`  
                                                                                                                                                                                                                                                                    `ccaGCCGACTGCCGACGAAGGGTCTACCTACACGGAAGACATTGGGCGCTTAAAGTTACGACGAGCGTAGGTTAACTACGAGAACTCTGGTACGTTTGACATTTATGTTGAGAACCAATCGTCTAACTGGAAGTACACAATGGCTGGTGCCCGATTAGGCTCTAACACTCTGAGGGATGGGAGACTGAACTTAGGGACCGGACAATATCGATTCCCTGTGGTTCGTAACGCCAAGTTCAACACTGTATACATCTTGTCAGATGAGACTACCCCTCTGAACATCATTGGGTGTGGCTGGGaa                                         <  1:823512/299‑1 (MQ=255)`  
                                                                                                                                                                                                                                                                     `aaGCAGACTGCCGACGACGGGTCTACCTCCACGGAAGACGTTGGGCGCTTACAGTTACTCCGAGCGTGGGGTAACTACGAGAACTATGGTACGTTTGACATTTATGTGGAGAACCAATCGACTAACTGGAAGTAAACAATGGCTTGTGCCCGATTAGGCTCTAACACTCTGAGGGCGGGGAGACTGAACTTAGGGAACGTAAAATATCGATTCCCTGTGGTTGGTAACGCCAAGTTCAACACTGGATACATCTTGTCAGAGGAGACTACCCCTCTGAACATCATTGGGTGTGGCTGGGAAg                                        <  1:174302/301‑1 (MQ=255)`  
                                                                                                                                                                                                                                                                              `gCCGACGACGGGTCTACCTCCACGGAAGACGTTGGGCGCTTACAGTTACGCCGAGCGTGGGTTAACTACGAGAACTCTGGTACGTTTGACATTTATGTTTAGAACAAATCGTCTAACTGGAAGTACACAATGGCTGGTGCCCGATTAGGATCTCACACACTGAGGGCGGGTAGACTGAACTTAGGGACCGGACAATAACGATTCCCTGTGGATGGTAAAGACAAGTTAAACACTGTATACAACTTGACAGATGAGACTACCCCGCTGAACATAATTGGGGGAGGCATGGAGAGTAactaca                               >  1:167622/1‑300 (MQ=255)`  
                                                                                                                                                                                                                                                                                        `ggTCTACCTCCACGGAAGACATTGGGCGCTTACAGTTACGCCGAGCGTTGGTTAACTACGAGAACTCTGGTACGTTTGACATTTATGTTGAGAACCAATCGTCTAACTGGAAGTACACAATGGCTGGTGCCCGATTATGCTCTAACACTCTGAGGGCTGGGATACTGAACTTAGGGACCGGACAATATCGATTCCCTGTGGTTGGTAACGCCAAGTTCAACACTGTATACATCTTGTCAGATTAGACTACCCCTCTGAACATCATTGGGTGTGGCTGGGAAGGTAACTACTTACGGAGAAg                     <  1:65144/301‑1 (MQ=255)`  
                                                                                                                                                                                                                                                                                        `ggTCTACCTCCACGGAAGACATTGGGCGCTTACAGTTACGCCGAGCGTGGGTTAACTACGAGAACTCTGGTACGTTTGACATTTATGTTGAGAACCAATCGTCTAACTGGAAGTACACAATGGCTGGTGCCAGATTAGGCTCTAACACTCTGAGGGCTGGTAGACTGAACTTCGAGATCGGACAATATCGATTCCCTGTGGTTAGTAACGCCATGTTCAACACTGTATACATCTTGTCAGATGCGACTACCCTTCTGAACATCATTGGgtgctgtctcttatatcactctccgtgcctact                     >  1:225650/1‑271 (MQ=255)`  
                                                                                                                                                                                                                                                                                                 `ccACGGAAGACATTGGGCGCTTACAGTTACGCCGAGCGTGGGTTAACTACGAGAACTCTGGTACGTTTGACATTTATGTTGAGAACCAATCGTCTAACTGGAAGTACACAATGGCTGGTGCCCGATTAGGCTCTAACACTCTGAGGGCTGGGAGACTGAACTTAGGGACCGGACAAGATCGATTCCCTGTGGTTGGTAACGCCAAGTTCAACACTGTATACATCTTGTCAGATGAGACTACCCCTCTGAACATCATTGGGTGTGGCTGGGAAGGTAACTACTTACGGAGAAGTTCCGGTAt            <  1:140574/301‑1 (MQ=255)`  
                                                                                                                                                                                                                                                                                                  `cACGGAAGACATTGGGCGCTTACAGTTACGCCGAGCGTGGGTTAACTACGAGAACTCTGGTACGTTTGACATTTATGTTGAGAACCAATCGTCTAACTGGAAGTACACAATGGCTGGTGCCCGATTAGGCTCTAACACTCTGAGGGCTGGGAGACTGAACTTAGGGACCGGACAATATCGATTCCCTGTGGTTGGTAACGCCAAGTTCAACACTGTATACATCTTGTCAGATGAGACTACCCCTCTGAACATCATTGGGTGTGGCTGGGAAGGTAACTACTTACGGAGAAGTTCCGGTAtc           >  1:376796/1‑300 (MQ=255)`  

                                                                                                                                                                                                                                                                                                            `|`                                                                                                                                                                                                                                                                                                             
`ATTCGATACACGATTCCTAGTGGAACATACAACGATGACACATTCACTACCTCTATTCATATTCCAACAATTTATGGTGCAAACTTCGGGAGGGGCAAAATCACTGTATTGGAGCCTGATGGTAAGATAACCGTGTTTGAGCAACCTACGGCTGGGTGGAATAGCGACCCTTGGCTGAGACTCAGCGGTAACTTGGAGGGACGCATGGTGTACATTGGGTTCAACATTAACTTCGTATATGAGTTCTCTAAGTTCCTCATCAAGCAGACTGCCGACGACGGGTCTACCTCCACGGAAGACATTGGGCGCTTACAGTTACGCCGAGCGTGGGTTAACTACGAGAACTCTGGTACGTTTGACATTTATGTTGAGAACCAATCGTCTAACTGGAAGTACACAATGGCTGGTGCCCGATTAGGCTCTAACACTCTGAGGGCTGGGAGACTGAACTTAGGGACCGGACAATATCGATTCCCTGTGGTTGGTAACGCCAAGTTCAACACTGTATACATCTTGTCAGATGAGACTACCCCTCTGAACATCATTGGGTGTGGCTGGGAAGGTAACTACTTACGGAGAAGTTCCGGTATTTAATTAAAT  >  NC_001604.1/26633‑27232`

  

Alignment Legend

Aligned base mismatch/match (shaded by quality score): `ATCG/ATCG` `< 3 ≤` `ATCG/ATCG` `< 13 ≤` `ATCG/ATCG` `< 26 ≤` `ATCG/ATCG` `< 36 ≤` `ATCG/ATCG` `< 38 ≤` `ATCG/ATCG`

Unaligned base: `atcg`    Masked matching base: `atcg`    Alignment gap: `‑`    Deleted base: `‑`
